# Evolutionary Changes in the Complexity of the Tectum of Nontetrapods: A Cladistic Approach

**DOI:** 10.1371/journal.pone.0003582

**Published:** 2008-10-30

**Authors:** Caio Maximino

**Affiliations:** Laboratory of Psychobiology and Experimental Psychopatology, Department of Psychology, Universidade Estadual Paulista, Bauru, Brazil; University of Maryland, United States of America

## Abstract

**Background:**

The tectum is a structure localized in the roof of the midbrain in vertebrates, and is taken to be highly conserved in evolution. The present article assessed three hypotheses concerning the evolution of lamination and citoarchitecture of the tectum of nontetrapod animals: 1) There is a significant degree of phylogenetic inertia in both traits studied (number of cellular layers and number of cell classes in tectum); 2) Both traits are positively correlated accross evolution after correction for phylogeny; and 3) Different developmental pathways should generate different patterns of lamination and cytoarchitecture.

**Methodology/Principal Findings:**

The hypotheses were tested using analytical-computational tools for phylogenetic hypothesis testing. Both traits presented a considerably large phylogenetic signal and were positively associated. However, no difference was found between two clades classified as per the general developmental pathways of their brains.

**Conclusions/Significance:**

The evidence amassed points to more variation in the tectum than would be expected by phylogeny in three species from the taxa analysed; this variation is not better explained by differences in the main course of development, as would be predicted by the developmental clade hypothesis. Those findings shed new light on the evolution of an functionally important structure in nontetrapods, the most basal radiations of vertebrates.

## Introduction

The tectum–a multisensory, topologically mapped structure in the roof of the midbrain–presents a remarkable degree of conservation in all vertebrate radiations [1]; although it varies in the extent of its development in different vertebrate classes, there is considerable evidence now to deem its layered structure, its cell types, and its hodological pattern as homologous in all vertebrates. In those vertebrates with a well-developed visual system, the tectum is dominated by retinal inputs; Huber and Crosby [2] demonstrated that “there exists a direct relation between the size of the eye and the development of certain layers of the of the optic tectum” (p. 15). The tectum is organized as a series of layers from its outer surface to a periventricular core that is present in all vertebrates with the exception of mammals and hagfishes. Each layer contains different neuronal classes, receives different kinds of sensory input, and projects to different neuronal centers, and can thus be understood as unique functional divisions [3]. Retinotectal projections terminate primarily in the outer layers, while somatosensory information is relayed primarily to deeper layers ([1]); both systems are mapped in register with each other within the tectum. As such, the major function of the tectum is “to localize a stimulus in space and to cause the animal to orient to the stimulus by moving its neck and/or its eyes” ([1], p. 311). Thus, even though extensive retinotectal projections in many vertebrates is taken as an evidence for a visual function of the tectum–to the point that it is often called “optic tectum”, even though the term is more appropriate for a retinorecipient region in this structure that is particularly dominating in visually-oriented animals –, one must notice that

“it is equally true that the tectum is a sensory correlation center and to a very considerable degree its size, and more particularly its lamination, evidences the relative variety and complexity of the non-optic afferent impulses reaching it from the brain stem and diencephalic region” (ref. [2], p. 15).

Tectal lamination shows increases and decreases across the different radiations in the vertebrate clade. Northcutt (ref. [3]) proposed the existence of a morphocline (the ordination of homologous character states in different taxa from primitive to derived states; cf. ref. [4]) of tectal laminae from hagfishes to amniotes. This is more clear in the morphocline from polypteriformes to teleosts, in which an increase in tectal lamination is observed, as well as in the coelacanth-lungfish-amphibian morphocline. However, there is a marked decrease in the number of tectal laminae in the latter, a case of phylogenetic reduction.

The tectum also presents many different cell types; the most commonly studied is the piriform cell, a pear-shaped neuron that have radially oriented neurons. This class of neurons is a common feature of tectal cytoarchitectural organization in vertebrates, with galeomorph sharks, skates and rays being the exception to the rule. Instead of those small piriform neurons, those cartilaginous fishes present a population of cells with a more elaborate dendritic profile [5], [6]. In teleost fishes, piriform, horizontal and multipolar tectal neurons are present, as well as a population of branched spiny neurons that appears to be homoplaseous to this radiation [1], [7]–[8]. Those cell types which constitute homoplasy in galeomorph sharks, skates, rays, and teleost fishes probably evolved independently ([1]), and are not present in nonteleost ray-finned fishes and squalomorph sharks [8]–[10]. The number of cell classes in the tectum, according to Northcutt (ref. [3]), can be ordinated in morphoclines, in the same way that was made with the number of layers. Variation is also observed in the hodology of the tectum; the nucleus isthmi, which presents reciprocal connections with the tectum in actinopterygii and tetrapods is not found in chondrichthyes or cyclostomes ([1]).

The developmental history of tectal organization is also of interest; in fact, frogs and fishes were the early model animals for the study of the development of retinotectal projections (eg. refs. [11]–[14]). Butler and Hodos (ref. [1]) proposed a taxonomy of brains that is derived from coarse-grained developmental histories, suggesting the existence of two types or organization:

We will define the first type as those species in which the brains are characterized by the neuronal cell bodies being unmigrated or only partially migrated away from the embryonic, periventricular matrix, which is the zone from which neurons develop (…). This pattern of organization will be referred to as **laminar**, in reference to the periventricular lamina in which the majority of neuronal cell bodies are located (…). Other species have brains in which extensive migration of neuronal cell bodies away from the periventricular matrix has occurred (…). The pattern of organization in brains with migration of the majority of neuronal cell bodies will be referred to as **elaborated** ([1], pp. 84–85; emphases in the original).

In the case of the tectum, these two developmental pathways are of direct interest, because this structure presents a core that is connected to the main ventricular system of the brain; its layered structure, as noted above, stems from this periventricular core. The different developmental pathways, thus, should be directly reflected in tectal lamination patterns.

It is difficult to discern the patterns of evolutionary change in tectal organization. Even though Northcutt (ref. [3]) proposed the existence of morphoclines for both number of layers and number of cell classes, no clear correlation between both traits can be recognized; this correlation is expected, since both morphological features provide for a high degree of precision in the spatial mapping function of the tectum. Ecomorphological theories (eg. refs [2], [ 15]–[17]) were proposed for evolutionary changes in the size of the tectum, usually relating the size of the eyes or the turbidity of watery environments with increases and decreases in this structure's relative size. Such analyses of adaptation are interesting *per se*, but a cladistic analysis of tectal morphology is still lacking. This type of analysis is complementary to researches on adaptation because it can point to patterns of brain evolution, such as concerted vs mosaic evolution (cf. ref. [18]), as well as to the extent in which a given trait presents a phylogenetic signal (a tendency “for evolutionarily related organisms to resemble each other, with no implication as to the mechanism that might cause such resemblance” [19]). Paraphrasing Northcutt (ref. [3]), adaptation studies describe the “why” of evolutionary changes, while cladistic studies describe the “what” of those changes. The present study attempts to describe the evolutionary changes in the number of tectal laminae and in the number of cell classes present in the tectum of nontetrapods of different clades (agnathans, actinopterygians, chondrochthyes and Dipnoi). It is hypothesised that, even though there is considerable variation in the state of those traits, a significant phylogenetic signal is present in both; it is proposed that a cladistic analysis is more parsimonious than the “morphocline” approach assumed by Northcutt (ref. [3]). Since evolutionary changes in organizational properties of brain areas tend to be concerted [18], an accessory hypothesis is made that a positive phylogenetic correlation should exist between both traits. It is also hypothesised that species with laminar and elaborated brains, as defined by Butler and Hodos (ref. [1]), should present differences in the pattern of lamination (and, if the accessory hypothesis is correct, also in the number of cell classes)–*viz*, those species which present elaborated brains should predictably have more layers in their tectum than species with laminar brains. In order to test those hypotheses, analytical-computational methods were used.

## Results

**Figure 1:**
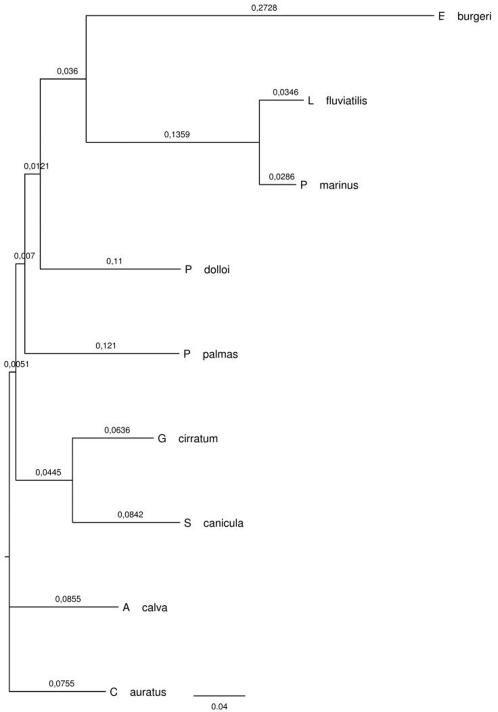
Phylogenetic relationships between species, as assessed by Neighborhood Joining of aligned cytochrome B sequences. Branch annotations refer to branch lengths.

**Figure 2:**
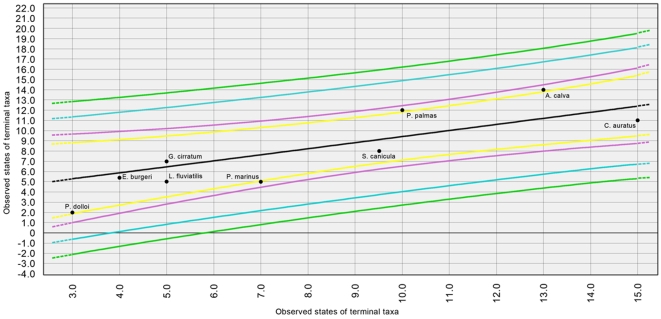
Prediction and confidence intervals for independent contrasts-based regression analysis. 90% and 95% prediction (orange and red, respectively) and 90% and 95% confidence (blue and orange) intervals are represented.

The estimated phylogenetic relations between species is presented in figure 1. Both traits presented a significant phylogenetic signal (table 1), and are positively associated (Felsenstein's correlation, *r* ^2^ = 0.654, *P* = 0.005, df = 6). Phylogenetic IC-based regressions yielded prediction and confidence intervals presented in figure 2. All species fell into the prediction intervals for the regression of number of cell classes into number of layers; the bowfin *Amia calva*, the lamprey *Petromyzon marinus* and the bichir *Polypterus palmas* fell outside of the 95% confidence interval for the regression. As such, those species present a higher (*A. calva* and *P. palmas*) or lower (*P. marinus*) number of cell classes in their tecta than would be expected by their pattern of lamination, as predicted to the taxa analysed.

**Table 1:** Results of the PHYSIG phylogenetic signal estimation.

Trait	Expected MSE_0_/MSE^1^	Observed MSE_0_/MSE	K^2^	Mean MSE permuted data	SD MSE permuted data	Skew MSE permuted data	P
Number of layers	−1.536	1.073	−6.987	41.558	16.273	2278.356	0.041
Number of cell types	−1.536	1.699	−1.106	36.583	15.117	2832.841	0.002

1 MSE_0_: Mean squared error of the tip data measured from the phylogenetically correct mean.

MSE: Mean squared error of the data calculated using the variance-covariance matrix derived from the phylogenetic tree.

2 K: the ratio between expected MSE0/MSE and observed MSE0/MSE with all the parameters set.

**Figure 3:**
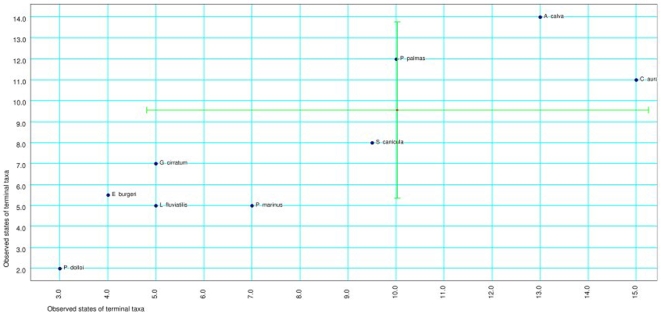
Root node reconstruction mapped back in the scatterplot for the relation between number of cell classes and number of layers in the tectum. Green lines represent 95% confidence intervals for the estimates of root node values.

Figure 3 presents the replotting of estimated ancestral states of both traits into the plot relating the observed values of terminal taxa, along with 95% confidence intervals for the estimates. Most species fall within the confidence intervals; the exceptions were *A.* calva (number of cell classes above upper CI for root node), *P. marinus* and *L. fluviatilis* (number of cell classes below lower CI for root node), *E. burgeri* (number of layers below lower CI for root node) and *P. dolloi* (both number of layers and number of cell classes below lower CI for root node). Since there was an association between the number of cell classes and the number of layers, those species which fall outside the 95% confidence intervals can be interpreted as having significantly departed from the common ancestor of the species studied in terms of the rules that should have governed this relationship.

**Figure 4:**
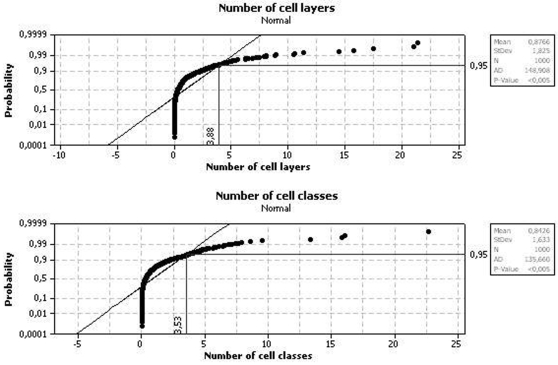
Anderson-Darling normality tests and hypothesis testing for the F-ratios in the simulated data set. Y-axis reference lines show the 95^th^-percentile, the critical value that demarcates statistical significance in differences between clades (see ref. [25]).

The phylogenetic ANOVA results are presented in figure 4 and table 2. No differences were found among both clades in terms of the organization and cytoarchitecture of their tecta.

**Table 2:** Analysis of variance comparing the number of laminae and number of cell classes in the tectum of type 1 (“laminar brains”) and type 2 (“elaborate brains”) species, as classified by Butler and Hodos (ref. [1]).

Source of variation	Sum of squares	df	Mean square	F	Conventional tabular		Simulation values	
					Critical value	P	Critical value	P
**Number of layers**								
Type of brain	1.3347	1	1.3347	0.0673	5.32	0.8019	3.88	0.91563
Error	138.8875	7	19.8411					
Total	140.2222	8	17.5278					
**Number of cell classes**								
Type of brain	0.1681	1	0.1681	0.0097	5.32	0.9240	3.53	0.9029
Error	121.3875	7	17.3411					
Total	121.5555	8	15.1944					

Critical values for *F* statistics and associated significance levels are presented for conventional tabular values (harmonic interpolation [26])–which would be appropriate only if the traits did not present a significant phylogenetic signal (see table 2) –, as well as based on analyses of data simulated along the phylogeny shown in figure 1 under a bounded Ornstein-Uhlenbeck model of trait change. The parameters for the OU model can be found in Table S1. See text for more information.

## Discussion

The present article tested three hypotheses regarding the phylogenetic history of tectal lamination and cytoarchitecture in the tectum of nontetrapods. A computational phylogenetic approach was used to test the phylogenetic signal, the association between traits, and differences among developmentally-classified clades in both characters. Each of those tests will be discussed in separate, and a general conclusion will be made latter.

There is a considerable degree of phylogenetic signal for both traits in the phylogeny studied. This is consistent with Blomberg et al.'s [19] observation that morphological variables tend to be less labile (ie, tend to present more phylogenetic inertia) than behavioral and physiological ones; this is ascertained by the fact that the use of 9 species in the phylogeny is sub-threshold for the detection of signal by Blomberg et al.'s [19] method. This also means that the data points analysed can not be considered to be independent, and phylogenetically-correct approaches must be used to understand them.

The IC-based approach for regression yielded results which are not compatible with Northcutt's (ref [3]) theory of morphoclines in vertebrate tectal organization. The number of cell layers, as well as the number of cell classes within the tectum, increased and decreased many times in the phylogeny. *A. calva* and *P. palmas* present a higher number of cell classes than would be expected if an increase in the number of laminae yields an increase in the number of cell classes in this phylogeny, while *P. marinus* present a slight tendency toward being outside the predicted relationship by having less cell classes than would be expected by the regression; however, all other species fall within the confidence and prediction intervals for the regression. This suggests that there is a direct relationship between both traits, and this is confirmed by the Felsenstein correlation coefficient for both variables. The analysis of the estimated ancestral state of both variables sheds more light on the problem: with the exception of *P. palmas* and *S. canicula*, no other species fall within the confidence intervals for the most basal node. This implies that there was considerable evolutionary change in the characters for the phylogeny considered, and the state of the traits studied is highly derived. In the cyprinid *Carassius auratus*, there was a proliferation of cell classes beyond the expectations of lamination. Thus, even though apomorphisms in cell types, for example, is observed in galeomorph sharks and teleosts [1], [5]–[8], only in the latter is the innovation accompanied by greater complexity of the tectum than expected by the phylogeny.

These differences are not best explained by the developmental pathways discerned by Butler and Hodos (ref. [1]), since both the bichir *P. palmas* and the bowfin *A. calva* are classified as having type 1 (“laminar”) brains, while *C. auratus* and *S. canicula* are classified as having type 2 (“elaborate”) brains. If anything, a trend towards greater lamination is found in *type 1* brains, instead of in type 2. It must be considered also that those species that present significant evolutionary changes in both traits are also those that present the higher degree of lamination and cell specialization in their groups. There is no reason to believe, however, that, within the taxa studied, any significant difference in the pattern of lamination and cytoarchitectural differentiation can be attributed to these coarse-grained developmental pathways.

The question of variety in the number of cell classes across species leaves open the more epistemological problem of which criteria are used to homologise cell groups across taxa. Developmental studies across taxa [35]–[42], either tetrapods and nontetrapods, suggest that the observable variety in tectal neuronal types results from the combined effects of somatic translocation, dendritic specialization and cell migration of a small set of 2–3 postmitotic cell forms; it has been proposed (Luis Puelles, personal communication, 21 Jul 2008) that this set is homologous in all vertebrates, while the secondary variants generated by the developmental processes delineated above are not. Nieuwenhuys and colleagues [43] reviewed the comparative literature on tectal cytoarchitecture and concluded that the laminar position of the somata of these secondary variants–as well as their dendrites' branching pattern–are highly apomorphic; thus, cell types which are considered homoplasic among taxa (based on topology) can, after consideration of relative birthdates, translocation patterns, hodology, neurochemical phenotypes, and gene expression patterns, be considered homologous. Thus, consideration of the identity of a given cell class in the tectum is tricky, since two species can have the same neuronal types in a different laminar arrangement.

Another caveat of the present analysis is the criteria for counting layers. In the literature on tectal lamination, it is usual to count plexiform layers along with cellular layers. However, as a consequence of differential cell migration, it is possible that the single superficial neuropil and the single periventricular stratum of those species which present least radial migration are (respectively) field homologous [44] to all plexiform layers and all cellular layers found in species with more elaborate cell migration (Luis Puelles, personal communication, 21 Jul 2008); if this hypothesis is proved to be true, both types of layers should be examined separately–not only on this article, but on comparative neuroanatomical studies in general.

Those caveats do not invalidate the hypotheses discussed and proposed in this article. The proposal of clades/grades of general developmental pathways is considerably hindered by the results found in the present work; as delineated in the last two paragraphs, the patterns of cell migration and differentiation in the optic tectum are considerably more complex than Butler's and Hodos' [1] hypothesis delineates. In fact, the differential morphogenetic pathways proposed by Nieuwenhuys et al. [43] and Puelles (pers. comm.) seen to be better predictors of morphological variability in adult tecta in different vertebrate species than the type I and type II general developmental processes proposed by Butler and Hodos; even though the main idea behind both hypotheses–cell migration patterns shape the evolutionary change of optic tectum complexity–is similar, one hypothesis is more complex than the other, and do not require that more inclusive taxa be distributed in grades according to morphogenetic pathways. These results are also consistent with the rejection of the morphocline hypothesis championed by Northcutt [3]; in conjunction with the morphogenetic hypothesis, as well as ecomorphological considerations (eg., refs. [16], [17]), the present data demonstrates that, in nontetrapods, tectal lamination and cell differentiation do not follow an evolutionary trend towards increased complexity. Rather, those traits probably involve a phylogenetically conserved component (the small set of postmitotic cells) as well as a more labile one, which could be the target of selective pressures. For example, in the lungfish *Protopterus dolloi*, a reduction in tectal complexity is probably related to the differential pressures of a (optically) less demanding environment (cf. Striedter's [18] discussion on brain size reduction in lungfishes). If morphogenetic units are indeed the link between genotype and phenotype [45], those selective pressures probably would cause reduced primary radial migration and nuclear translocation (or even secondary perikaryal translocations), which would in turn account for the reduced complexity in the tectum opticum of these species.

The proposed morphogenetic framework can also explain the phylogenetic correlation between structure lamination and cell differentiation found in the present article. Regression analyses were not undertaken in this article because a causal, directional relationship between layering and cell differentiation was not assumed. The correlation between those two morphological traits is best explained, however, by a framework which relates both to differential migration patterns. If, as proposed, the observable variability in cell classes is a function of translocation, dendritic specialization and migration of a small set of (field homologous) pioneer cells, and radial migration of cells is responsible for producing the layered pattern, then the positive correlation between the number of cell classes and the number of layers can be explained by a third (causal) variable–*viz*, cell migration processes.

The hypothesis that complex cell migration patterns make up the causal link between changes in the number of layers and the number of cell classes in the tectal formation ought to be further explored in developmental comparative studies. For example, the role of cadherins [46] in this process could be investigated, or the phylogenetic distribution of cadherin subtypes [47] related to the morphological data on tectal complexity. A full account of the evolution of tectal lamination in vertebrates is still needed, but researchers would benefit from a developmental, comparative and ecomorphological approach.

## Materials and Methods

### Data set

The data set was obtained from a review in the literature. Table 3 presents the species analysed, number of layers in the tectum and number of tectal cell classes for each of the species, as well as the reference from where the data was obtained. Only cellular laminae were considered in the present work, the cell classes were obtained from each paper. Classification of brains between type I and type II follow that proposed by Butler and Hodos (ref. [1]), pp. 84–89 (cf. their table 4-1).

**Table 3:** Summary for the values of the traits, separated by species and clade.

Clade (developmental classification)	Species	Trait 1: Number of layers in tectum	Trait 2: Number of cell classes in tectum	Reference
Type 1	River lamprey, *Lampreta fluviatilis*	5	5	[28]
	Marine lamprey, *Petromyzon marinus*	7	5	[29]
	Bichir, *Polypterus palmas*	10	12	[8]
	Bowfin, *Amia calva*	13	14	[8]
	African lungfish, *Protopterus dolloi*	3	2	[30]
Type 2	Hagfish, *Eptatretus burgeri*	4	5	[31]
	Nurse shark, *Ginglymostoma cirratum*	5	7	[6]
	Chain dogfish, *Scyliorhinus canicula*	9	8	[5], [ 9]–[10], [32]
	Goldfish, *Carassius auratus*	15	11	[7]–[8], [ 33]–[34]

“Type 1” and “Type 2” refer to the classification of laminar and elaborated brains proposed by Butler and Hodos (ref. [1]).

### Phylogenetic distances

Based on common phylogenetic data, a tree was constructed using the Mesquite software [20]. Phylogenetic distances were estimated using cytochrome B sequence data for the species chosen (GenBank accession numbers: NC_002079.1, NC_001626.1, NC_001131.1, NC_004742.1, NC_001778.1, NC_001950.1, NC_002807.1) by the Neighborhood Joining method [21]. Sequences were aligned using ClustalW [22] and processed into a phylogenetic variance-covariance matrix using the “Export Distance Matrix” function of Mesquite. This matrix was latter used for estimation of phylogenetic signal.

### Estimation of phylogenetic signal in both traits

In order to estimate whether traits 1 (number of layers in tectum) and 2 (number of tectal cell classes) presented phylogenetic signal, the PHYSIG algorithm [19] was applied. A randomization test was applied in the data for the two traits, using the PHYSIG.M Matlab script [19], which computes a K statistic to gauge how much phylogenetic signal is present, as well as presenting p-values for this statistic. Distance matrices were altered by using an Ornstein-Uhlenbeck model of trait evolution with the *d* parameter set to 1.005, thus making the tree more hierarchical. 1000 permutations were used to estimate *K* and generate the associated p-values.

### Phylogenetically Independent Contrasts

Felsenstein's independent contrasts (IC [23]) were used to predict taxon-specific changes in each of the traits from a phylogenetically-correct regression approach. Independent contrasts are calculated as differences in the value of a trait between two sister species divided by the square root of the sum of their branch lengths. Garland Jr. and Ives [24] proposed that the formulation of regression in terms of independent contrasts is possible by the removal of the constant coefficient and the reformulation of independent contrasts in order to generate confidence and prediction intervals. For this, the PDTREE [24], [25] package of PDAP was used.

### Comparison between type 1 and type 2 brain clades

To assess whether animals that possessed type 1 and type 2 brains differed in the number of tectal layers or in the number of cell classes, a phylogenetic approach to ANOVA was used [25]. For this, the PDSIMUL package of PDAP [25] was used to generate 1000 Monte Carlo simulations of phylogeny-weighted tip values as a null empirical distribution. A bounded Gradual Ornstein-Uhlenbeck model of trait evolution [25] was assumed, and parameters can be found in Supplemental Material 1. For each of the simulated values, the PDANOVA package of PDAP (Garland Jr et al., 1993) was used to compute within- and between-group sums of squares, mean squares, and corresponding F ratios, as in conventional ANOVA. MINITAB 14.1 was then used to compute the 95^th^ percentile of the F-ratio distribution. Following Garland Jr et al. (1993), if the F ratio for the real data set (obtained from the PDSINGLE package of PDAP; Garland et al., 1993) exceeds the upper 95^th^ percentile of the empirical null distribution, it should be concluded that the two clades (type 1 and type 2) differe significantly in the traits. Critical values were generated by harmonic interpolation [27] using the StaTable 1.0.1 software.

## Supporting Information

Table S1 Parameters of the Ornstein-Uhlenbeck simulation model. Parameters used in the Ornstein-Uhlenbeck model that simulated the evolution of the traits studied, for latter use as null distributions in Phylogenetic ANOVAs.
(0.08 MB DOC)
