# Supplementary material for: Evolutionary Changes in the Complexity of the Tectum of Nontetrapods: A Cladistic Approach
Source: PLoS One. 2008 Oct 30;3(10):e3582. doi: 10.1371/journal.pone.0003582 (PMC2571994; doi:10.1371/journal.pone.0003582)
Supplement: Table S1 — Parameters of the Ornstein-Uhlenbeck simulation model. Parameters used in the Ornstein-Uhlenbeck model that simulated the evolution of the traits studied, for latter use as null distributions in Phylogenetic ANOVAs. (0.08 MB DOC) [file pone.0003582.s001.doc]

**Table S1 –** Parameters of the Ornstein-Uhlenbeck simulation model.

| Parameter | Referent | Value |
| --- | --- | --- |
| Initial values | Trait 1 | 7.9444 |
|  | Trait 2 | 7.7222 |
|  | Correlation | 0.8721 |
| Selected tip variance | Trait 1 | 17.5278 |
|  | Trait 2 | 15.1944 |
| OU decay constants | Trait 1 | 0.0000001 |
|  | Trait 2 | 0.0000001 |
| OU starting peak value | Trait 1 | 7.9444 |
|  | Trait 2 | 7.7222 |
| Speed of change of peak | Trait 1 | 0.0000002 |
|  | Trait 2 | 0.0000002 |
| Variance of peak change | Trait 1 | 0.00000002 |
|  | Trait 2 | 0.00000002 |
| Upper bounds | Trait 1 | 15.0 |
|  | Trait 2 | 12.0 |
| Lower bounds | Trait 1 | 1.0 |
|  | Trait 2 | 1.0 |
